# Tenecteplase With or Without Mechanical Thrombectomy in Acute Ischemic Stroke at 4.5 to 24 h: An Updated Meta-Analysis of Randomized Controlled Trials

**DOI:** 10.3390/neurolint18060116

**Published:** 2026-06-11

**Authors:** Beatrice Dell’Acqua, Carmelina Maria Costa, Andrea Cerri, Alessandro Francia, Simone Vidale

**Affiliations:** 1 Department of Neurology & Stroke Unit, ASST Sette Laghi, 21100 Varese, Italy; 2 Department of Neurology & Stroke Unit, Bicocca University, 20126 Milano, Italy

**Keywords:** Tenecteplase, mechanical thrombectomy, intravenous thrombolysis, 4.5–24 h-extended window

## Abstract

Background and Purpose: Tenecteplase (TNK) within 4.5 h from symptom onset is not inferior to alteplase in treating ischemic stroke. In recent years, some randomized controlled trials (RCTs) have investigated the efficacy of extending the therapeutic window up to 24 h. This updated meta-analysis aims to synthesize the results of these RCTs comparing TNK to the best medical treatment (BMT) with or without endovascular thrombectomy. Methods: In accordance with PRISMA guidelines, all RCTs comparing TNK with BMT in adult patients between 4.5 and 24 h were systematically searched. The primary endpoint was good functional outcome at 90 days (mRS 0–2). Secondary endpoints included excellent outcome (mRS 0–1), symptomatic intracerebral hemorrhage (sICH), 90-day mortality, complete reperfusion at 24 h. Odd and Hazard ratios (ORs; HRs) were pooled using meta-analytic methods. Results: A total of seven RCTs involving 1754 patients were included. The rates of the primary endpoint were higher in TNK-treated patients (HR: 1.15; 95% CI: 1.03–1.27), as were rates of excellent functional outcome (HR: 1.29; 95% CI: 1.08–1.55). In the subgroup receiving intravenous therapy (IVT) alone, the primary endpoint was significantly more frequent in the TNK group than in the BMT group (OR: 1.47; 95% CI: 1.17–1.84; *p* for heterogeneity < 0.0001). TNK treatment was also associated with higher reperfusion rates compared with BMT, reflecting a greater proportion of saved ischemic penumbra as assessed via perfusion imaging. Although symptomatic intracranial hemorrhage (sICH) occurred more frequently in TNK-treated patients, the difference did not reach statistical significance, and mortality rates were comparable between treatment groups. Conclusions: Tenecteplase administered between 4.5 and 24 h is associated with improved rates of both good and excellent functional outcomes compared with BMT, especially in patients treated with IVT alone. Additionally, TNK is linked to higher rates of reperfusion.

## 1. Introduction

Acute ischemic stroke (AIS) represents a leading contributor to global morbidity and mortality, IVT continues to be the cornerstone of medical therapy for eligible patients [1].

TNK, a bioengineered variant of human tissue plasminogen activator, has demonstrated non-inferiority to alteplase—and potential superiority in certain contexts—when administered within 4.5 h of symptom onset in acute ischemic stroke [2].

In recent years, multiple randomized controlled trials have evaluated the comparative safety and efficacy of TNK versus alteplase in patients with acute ischemic stroke treated within 4.5 h hours of symptom onset [3,4,5,6,7,8,9,10,11,12,13]. These studies collectively suggest that TNK, when administered as an acute reperfusion therapy for ischemic stroke within the conventional therapeutic window and at the standard dose of 0.25 mg/kg, is associated with a numerically higher rate of excellent functional outcomes at 3 months—defined as a modified Rankin Scale (mRS) score of 0–1—compared with alteplase. Beyond this favourable efficacy signal, TNK may also provide several practical and clinical advantages that are highly relevant in the acute stroke setting. In particular, its single-bolus administration, as opposed to the continuous infusion required for alteplase, allows for more rapid and simplified delivery, which can translate into shorter door-to-needle times and improved workflow efficiency in emergency settings. Furthermore, TNK has been associated with higher rates of early arterial recanalization, especially in patients with large vessel occlusion, potentially facilitating faster restoration of cerebral blood flow. This enhanced reperfusion capability may also contribute to a reduction in perfusion deficit volumes, reflecting a greater preservation of ischemic penumbra and, ultimately, improved neurological outcomes. TNK is a genetically modified variant of alteplase characterized by a longer elimination half-life, allowing for single-bolus administration, and a higher fibrin specificity, which enhances its affinity for fibrin-bound plasminogen at the site of thrombus. However, its increased fibrin specificity and prolonged circulation have raised theoretical concerns regarding potential off-target thrombolytic activity and bleeding risk, including interaction with circulating fibrin degradation products, without a significant increased incidence of systemic or intracranial haemorrhagic complications compared with alteplase [3], supporting a favourable safety profile in acute ischemic stroke [2]. Taken together, these pharmacological and logistical advantages position TNK as an increasingly attractive alternative to alteplase in the management of acute ischemic stroke, with potential implications for both clinical efficacy and optimization of stroke care systems. Given that endovascular thrombectomy (EVT) performed within 24 h of symptom onset has proven to be effective for selected patients with acute large vessel occlusion stroke [14,15,16,17], in recent years, multiple studies have investigated the feasibility and efficacy of also extending the therapeutic window for thrombolytic treatments, including TNK [18,19,20,21,22,23,24].

Within the subgroup of patients who did not have access to EVT, TNK was associated with significant gains across multiple outcomes, including excellent and good functional outcome, rates of recanalization, and early neurological improvement. In contrast, among patients in the EVT-permitted subgroup, the benefit of TNK was largely restricted to enhanced vessel recanalization, with no significant impact on functional or neurological endpoints [25].

Given that non-large vessel occlusion acute ischemic stroke is more prevalent than stroke due to large-vessel occlusion, extending the therapeutic window for intravenous thrombolysis up to 24 h could represent a major shift in treatment paradigms. Such an approach may allow a greater proportion of patients to receive acute reperfusion therapy, with the potential to improve clinical outcomes and reduce the overall societal burden of ischemic stroke.

For these reasons, we conducted an updated pooled analysis of previous randomized controlled trials investigating the efficacy and safety of TNK administration within the extended therapeutic window.

## 2. Materials and Methods

**Figure 1:**
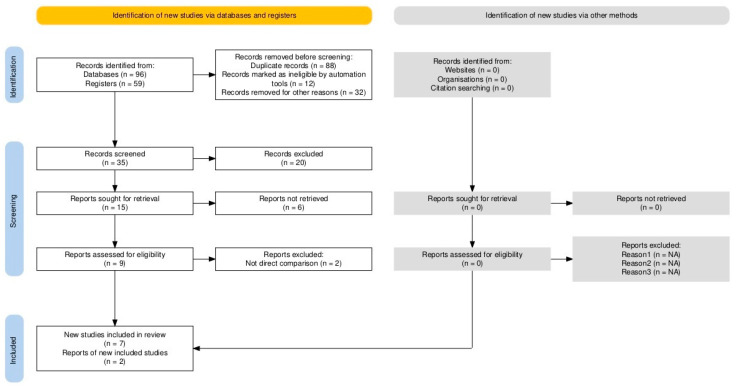
PRISMA flowchart illustrating the study selection process, including the number of records identified through database searching, records screened, full-text articles assessed for eligibility, and studies included in the meta-analysis, with reasons for exclusions at each stage.

This systematic review and meta-analysis were performed in accordance with the guidelines outlined by the Preferred Reporting Items for Systematic Reviews and Meta-Analyses (PRISMA), following the PRISMA checklist (supplementary Table S1). The protocol has been registered in the International Prospective Register of Systematic Reviews (registration ID: CRD420261351169). The selection process is illustrated as a PRISMA flow diagram (Figure 1). Data extraction and statistical analyses were performed using Review Manager (version 5.3, The Cochrane Col-laboration 2012, Copenhagen, Denmark) and Meta Software version 2.0.

Two independent reviewers conducted a comprehensive literature search in PubMed, EMBASE, and the Cochrane Central Register of Controlled Trials for studies comparing Tenecteplase at a standard dosage of 0.25 mg/Kg (TNK) versus BMT, including alteplase administration, all over the world. Patients could also be treated with mechanical thrombectomy in both groups, if indicated. Our search history was between January 2013 and March 2026. The search strategy employed a combination of keywords and MeSH terms, including “Ischemic Stroke”, “Tenecteplase”, “late window”, and “extended time window”. The full Boolean syntaxis for the literature search is reported in the Supplemental Materials.

Because this is a pooled analysis of aggregated data from previous published randomized controlled trials, neither ethical approval nor informed consent was required.

### 2.1. Selection Criteria

This meta-analysis considered only randomized controlled trials that assessed the clinical efficacy and safety of TNK vs. BMT in adults (aged ≥ 18 years) who experienced acute ischemic stroke with symptoms onset beyond 4.5 h. Only studies published in English were included. Case reports, small case series (fewer than 20 patients), conference abstracts, and review article were excluded. Patients in the interventional arm received TNK with or without adjunctive EVT, whereas the control group consisted of those treated with the best medical treatment, including alteplase, with or without EVT, according to the ESO-ESMINT (2019), and AHA/ASA (2019) guidelines [1,26]. Two reviewers independently extracted data on baseline characteristics and outcomes for each study. The methodological quality of the included trials was assessed using ROB2 tools [27].

### 2.2. Endpoints

The primary endpoint was represented by good functional outcome, defined as a mRS score below 3 at 90 days post-stroke. Secondary endpoints for efficacy included the excellent functional outcome at 3-month follow-up (mRS < 2) and reperfusion state, defined as the reduction of more than 90% of the penumbra at 24 h neuroimaging control from baseline, while safety endpoints were mortality at 90 days from stroke and occurrence of sICH. We reported the lack of data on outcome, when appropriate. The primary and secondary endpoints regarding functional outcome were investigated by subgrouping studies by type of treatment—specifically IVT-only and bridging therapy—when data were available.

### 2.3. Statistical Analysis

We performed a statistical analysis on the pooling data from the intervention and the control group. Outcome heterogeneity was evaluated with Cochrane’s Q test and I2. An overall *p* value of <0.05 was considered statistically significant. Random-effects models were applied to reduce the risks of confounders influence with the inverse variance method. Odd and Hazard ratios (ORs; HRs) and 95% confidence interval (CI) values were calculated using the DerSimonian & Laird model for primary and secondary endpoints. We reported the analysis results graphically using forest plots for outcomes of single included trials and the total treatment effects. We also reported funnel plots for the analysis regarding the functional outcomes to report potential bias in the included studies for each functional outcome endpoint. Data analyses were performed using Review Manager (version 5.3, The Cochrane Collaboration 2012, Copenhagen, Denmark) and Meta Software version 2.0.

## 3. Results

The pooled analysis included seven studies with a total of 1754 patients. The baseline characteristics of the seven RCT studies included are reported in Table 1. Interventional and control groups were equally distributed with 860 and 894 patients treated with TNK (49%) and BMT (51%), respectively. Five trials were conducted in China, while two were conducted in Australia and U.S. and Canada. Considering differences in country, the main ethnic group included in the pooled analysis was Asian, with 1506 patients. The median age of the entire sample was 65.6 years, and the patients included in the analysis were mainly males (62.3%). The median NIHSS score at admission was 8. The median volumes of the ischemic core and hypoperfused lesion were 5.3 mL and 90.4 mL, respectively. Table 2 shows differences in the main baseline characteristics of the entire sample between groups. The methodological quality of the included trials excluded risk of bias for all studies, except for two (ROB2 plot reported in Figure S1 [28]).

### 3.1. Primary Endpoint

**Figure 2:**
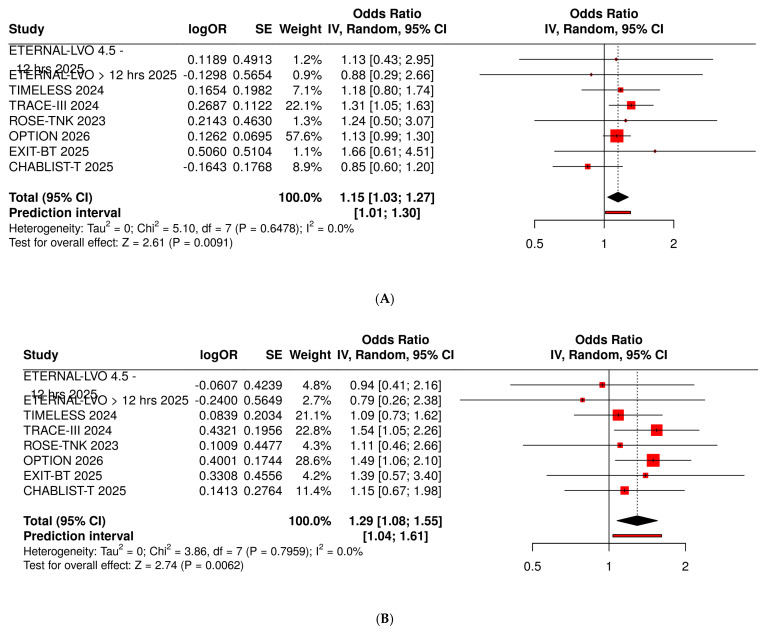
(**A**) Forest plot of pooled analysis of primary endpoint distributed by study type (TNK vs. BMT), showing the primary endpoint results, (good functional outcome) stratified by study type, comparing TNK versus BMT, with the OR for each study. (**B**) Forest plot of excellent functional outcome (secondary endopoint), displaying the effect estimates for excellent functional outcome across included studies comparing TNK vs. BMT, with individual study results and the overall pooled effect size with the ORs. [18,19,20,21,22,23,24].

**Figure 3:**
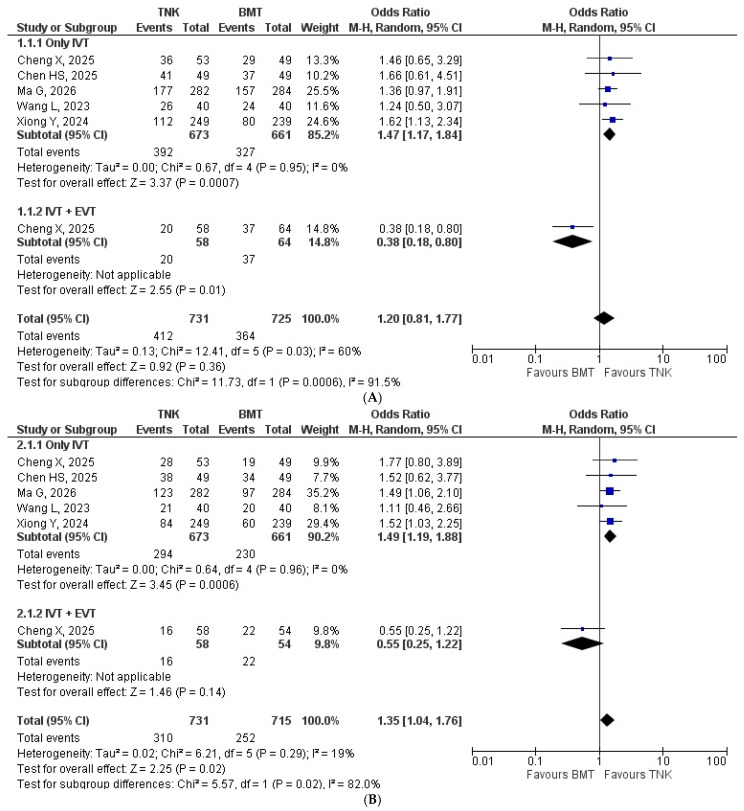
(**A**) Forest plot of good functional outcome in IVT-only therapy and bridging therapy (BT) across included studies comparing TNK vs. BMT, showing individual study effect estimates and the pooled overall effect size with corresponding confidence intervals. (**B**) Forest plot of excellent functional outcome in IVT-only therapy and BT across included studies comparing TNK vs. BMT, showing individual study effect estimates and the pooled overall effect size with corresponding confidence intervals. [18,19,20,21,22,23,24].

The rates of good functional outcome were globally and significantly higher in TNK treated patients (OR: 1.15; 95%CI: 1.03–1.27; *p*_heterogeneity_ < 0.0001). A forest plot of this pooled analysis is presented in Figure 2A. In the subgroup of the subjects with only IVT therapy, a good functional outcome was observed in 58.2% and 49.5% of TNK and BMT groups, respectively (OR: 1.47; 95%CI: 1.17–1.84; *p*_heterogeneity_ < 0.0001). A forest plot of this analysis is presented in Figure 3A. In the bridging therapy subgroup, a higher rate of good functional outcome was achieved in patients with BMT (Figure 3A).

### 3.2. Secondary Endpoints

An excellent functional outcome was reached with higher probability in patients treated with TNK than BMT with a rate of 29% (OR: 1.29; 95%CI: 1.08–1.55; *p*_heterogeneity_ 0.7959). A forest plot of this analysis is presented in Figure 2B. In patients treated only with IVT, an excellent functional outcome was observed in 43.7% and 34.8% of patients in the TNK and BMT groups, respectively (OR: 1.49; 95%CI: 1.19–1.88; *p*_heterogeneity_ 0.96). In the group of subjects treated with BT, the rate of achieved endpoints was higher in the BMT group without statistical significance (OR: 0.55; 95%CI: 0.25–1.22). A forest plot of this subgroup analysis is presented in Figure 3B. Reperfusion was observed in 36.2% and 28% of patients treated with TNK and BMT, respectively, with significant statistical difference between groups (OR: 1.72: 95%CI; 1.06–2.79; *p*_heterogeneity_ 0.0.01). The distribution by type of treatment is represented in the forest plot reported in Figure S2 of the Supplemental Materials. Considering safety endpoints, the 90-day mortality rate was relatively balanced between groups with 11.2% and 9.9% in the TNK and BMT groups, respectively (OR: 1.13; 95%CI: 0.84–1.52; *p*_heterogeneity_ 0.78—Figure S3), while ICH occurred with higher probability in the TNK-treated patients, without statistical significance between the interventional and control groups (3.1% versus 1.3%; OR: 2.03; 95%CI: 0.97–4.26; *p*_heterogeneity_ 0.37—Figure S4).

## 4. Discussion and Conclusions

This systematic review of seven RCT studies [18,19,20,21,22,23,24] found that TNK was associated with a statistically significant improvement in functional outcomes (90-day mRS: 0–2) compared to BMT in patients with acute ischemic stroke treated within the extended therapeutic window (4.5–24 h), suggesting a clinically meaningful benefit of TNK that supports its role as an effective reperfusion strategy beyond the conventional 4.5 h window. In trials conducted to date, both patients who did not undergo EVT (e.g., TRACE-III, ETERNAL-LVO, OPTION, ROSE-TNK, EXIT-BT) [18,19,20,22,24] and those treated with IVT with the possibility of subsequent EVT (e.g., TIMELESS, CHABLIS-T II) [21,23] were enrolled. A recent meta-analysis [29] evaluating TNK versus BMT or placebo in adults treated 4.5 to 24 h after acute ischemic stroke incorporated a prespecified stratification based on EVT availability (EVT-unavailable vs. EVT-eligible populations). This approach enabled a more precise assessment of treatment effects across differing clinical contexts and showed that the relative benefit of TNK was more pronounced in settings where EVT was not available, supporting its use as a viable reperfusion option in resource-constrained environments.

Among the seven randomized controlled trials included in the meta-analysis, the more favourable outcomes were observed in TRACE-III and OPTION. This might be explained by distinct but complementary selection mechanisms rather than a uniform treatment effect across trials. OPTION enrolled a highly selected population with minimal baseline ischemic injury and exclusion of large vessel occlusion, resulting in near-zero ischemic core volumes and an intrinsically high probability of good functional outcomes, potentially leading to a ceiling effect. In contrast, TRACE-III included patients with intermediate infarct core volumes but very large perfusion lesion volumes, indicating a substantial ischemic penumbra. This imaging profile suggests the presence of viable but hypoperfused tissue and preserved collateral circulation, identifying patients with a high potential for tissue salvage despite delayed presentation. Taken together, these differences suggest that OPTION reflects a “low-baseline-injury” population with inherently favourable prognosis, whereas TRACE-III represents a “high-salvage-potential” population characterized by a large mismatch profile. These complementary mechanisms likely contributed to the more pronounced treatment effects observed in these two trials compared with the remaining studies, which enrolled more heterogeneous populations with varying infarct burden, occlusion patterns, and imaging selection criteria.

The time to randomization might be another determinant of thrombolysis efficacy in acute ischemic stroke, as longer delays are associated with progressive infarct growth, reduction in salvageable penumbra, and a higher likelihood of futile reperfusion. So we evaluated whether time to randomization may have influenced the more favourable outcomes observed in these two studies. However, in OPTION, although the median onset-to-randomization time was approximately 12.4 h, the observed outcomes were likely driven primarily by highly restrictive imaging selection, resulting in minimal baseline ischemic injury (near-zero core volumes). This suggests that the treatment effect was largely influenced by patient selection rather than timing alone. Similarly, in TRACE-III, while a substantial proportion of patients were treated within 9 h (~33.9%), the study population was characterized by a pronounced perfusion–core mismatch, with relatively small infarct cores and very large perfusion lesions. This indicates substantial salvageable tissue and preserved collateral circulation, which are strong predictors of response to reperfusion therapies, even beyond the influence of time alone. In contrast, other included trials exhibited greater heterogeneity in baseline imaging profiles, vascular occlusion patterns, and eligibility criteria, which may have diluted the observable treatment effect. This variability likely reflects differences in infarct biology, collateral status, and patient selection strategies across studies, thereby limiting the ability to identify a uniform treatment effect across the entire pooled population. Future studies should aim to reduce such heterogeneity by adopting more standardized imaging protocols, harmonized definitions of ischemic core and penumbra, and consistent thresholds for treatment eligibility across different platforms and software packages. In addition, stratification by key prognostic variables—such as occlusion location, baseline infarct core volume, collateral circulation status, and onset-to-randomization time—may help us better delineate subgroups most likely to benefit from tenecteplase. Finally, future randomized trials with prespecified imaging-based enrichment strategies and uniform core laboratory adjudication could further improve comparability between studies and strengthen the precision of pooled estimates in subsequent meta-analyses.

Considering the subgroup analyses in patients treated with IVT alone, without EVT, TNK was associated with a substantially higher rate of good functional outcomes compared with BMT. This finding reinforces the hypothesis that TNK may be particularly advantageous in settings where EVT is not performed or not available. The observed effect may be explained by TNK’s properties, including fibrin specificity, longer half-life, and the fastest administration technique, which may facilitate treatment initiation and improved early reperfusion.

In the BT subgroup—where patients were eligible for EVT or subsequently underwent EVT—the benefit of TNK on functional outcomes appeared attenuated, with better results observed in the BMT group. This apparent discrepancy may reflect the dominant effect of EVT in determining clinical outcomes, so that any incremental benefit of TNK may be masked by the efficacy of EVT, limiting its observable impact. The differential use of endovascular thrombectomy (EVT) across the included studies may represent an additional source of clinical heterogeneity potentially influencing both primary and secondary endpoints. In particular, patients undergoing bridging therapy (IVT plus EVT) are typically characterized by different baseline clinical and radiological profiles compared with patients treated with intravenous thrombolysis (IVT) alone, including higher rates of large-vessel occlusion, greater stroke severity, and distinct reperfusion dynamics. Moreover, the strong therapeutic effect of EVT itself may partially attenuate or mask the incremental contribution of tenecteplase on functional and imaging outcomes, thereby reducing the ability to detect treatment-related differences between groups. However, the overall proportion of patients treated with EVT across the included trials was relatively limited compared with those receiving IVT alone, which likely mitigated the magnitude of its influence on the pooled estimates. Furthermore, subgroup analyses stratified by treatment strategy were performed to partially account for this variability. Nevertheless, residual heterogeneity related to differences in EVT eligibility criteria, procedural timing, reperfusion success rates, and local thrombectomy workflows cannot be entirely excluded and should be considered when interpreting the results.

Regarding the analysis of secondary endpoints TNK was associated with a significantly higher probability of achieving an excellent functional outcome (90-day mRS 0–1) compared with BMT. This finding reinforces the notion that TNK may not only improve general functional recovery but also increase the likelihood of near-complete neurological restitution, which represents a particularly meaningful outcome from both patient-centred and health system perspectives. This effect was even more pronounced in the subgroup of patients treated with IVT alone, where TNK was associated with a markedly higher proportion of excellent outcomes compared with BMT, as also reported in a recent meta-analysis [29]. These results are in line with the primary analysis and suggest that, in the absence of EVT, TNK may exert its maximal therapeutic potential. Aligned with the results shown above, also regarding excellent functional outcome, in the bridging therapy subgroup, a lower rate of excellent functional outcome was observed in the TNK group compared with BMT, although this difference did not reach statistical significance.

TNK demonstrated a significant advantage in terms of reperfusion rates compared with BMT, defined as a reduction of more than 90% of the penumbra volume at 24 h neuroimaging follow-up compared with baseline. However, this finding should be interpreted with caution given the substantial heterogeneity in the definition and assessment of reperfusion across the included studies. As highlighted above, the trials adopted different imaging modalities and criteria to define salvageable tissue and reperfusion, including CT perfusion (CTP), diffusion-weighted imaging (DWI), and perfusion–diffusion MRI, analyzed through different automated software platforms such as RAPID, 3D-Slicer, CTPdoc, and AutoMIStar. In addition, the thresholds used to define ischemic core and penumbra varied considerably between studies, including differences in relative cerebral blood flow cut-offs (<30%), Tmax thresholds (>6 s), mismatch ratios (>1.2 to >1.8), and core volume limits (<50 mL to <70 mL). These methodological inconsistencies, together with variability in imaging timing and operational definitions of reperfusion, may have influenced the assessment of penumbral salvage and contributed to residual heterogeneity across studies, thereby limiting the comparability and precision of pooled estimates for this imaging-derived secondary endpoint.

Regarding safety outcomes, no significant differences were observed in 90-day mortality between the TNK and BMT groups, suggesting that TNK does not confer an increased risk of death. However, there was a numerically higher incidence of sICH in patients treated with TNK, although this did not reach statistical significance and remains within an acceptable range consistent with the known risk profile of thrombolytic therapies.

An important aspect—and a limitation—of the present analysis pertains to the geographic distribution of the included trials and, consequently, the ethnic composition of the study population. Specifically, five out of the seven randomized controlled trials were conducted in China, whereas only two studies were performed in Western settings (Australia and North America). As a result, the pooled cohort is largely composed of Asian patients, leading to a substantial imbalance in population representation.

This predominance of an ethnic group warrants careful consideration when interpreting the findings, particularly with respect to their external validity and generalizability to more diverse, global populations. Ethnic and regional differences are known to play a significant role in stroke epidemiology, including variations in the prevalence and distribution of vascular risk factors, underlying stroke mechanisms, and access to acute stroke care. These factors may, in turn, influence both the biological response to reperfusion therapies and overall clinical outcomes.

Asian populations have consistently been reported to have a higher prevalence of intracranial atherosclerotic disease, as well as distinct patterns of vascular occlusion compared with Western populations, in which extracranial atherosclerosis and cardioembolic aetiologies are more commonly observed. Such pathophysiological differences may impact clot composition, collateral circulation, and responsiveness to thrombolytic agents, potentially modifying the efficacy profile of TNK.

Moreover, differences in healthcare infrastructure, stroke pathways, imaging selection criteria, and time-to-treatment metrics across regions may further contribute to heterogeneity in treatment effects. Altogether, these considerations underscore the need for caution in extrapolating the present results to non-Asian populations and highlight the importance of conducting further large-scale, multicenter trials in more ethnically and geographically diverse cohorts to validate and refine these findings. Overall, although the present findings provide meaningful and clinically relevant evidence supporting both the efficacy and safety of TNK in the extended therapeutic window, the marked predominance of Asian patients in the pooled population highlights an important limitation in terms of external validity. This imbalance reinforces the need for further large-scale, rigorously designed, multicenter randomized controlled trials enrolling more ethnically and geographically diverse populations.

Such studies are crucial not only to confirm the reproducibility and generalizability of these results across different demographic groups but also to better characterize potential variations in treatment response related to genetic background, stroke etiology, and healthcare system organization. Moreover, expanding the evidence base to include a broader spectrum of clinical settings—ranging from high-resource comprehensive stroke centres to more resource-limited environments—will be essential to define the optimal role of TNK in contemporary stroke care pathways. In this context, ongoing trials such as TENACITY (ClinicalTrials.gov ID: NCT07361302) are expected to provide additional high-quality data that may help clarify the efficacy and safety profile of TNK in diverse patient populations, further informing clinical decision-making and potentially contributing to future guideline recommendations. Another limitation of the current meta-analysis is the study level methodology that could limit our results. Study-level methodology limits the ability to perform more refined adjustments for confounding variables, explore interactions across clinically relevant subgroups, or account for heterogeneity in baseline characteristics such as age, stroke severity, imaging selection criteria, and time-to-treatment intervals. As a result, the observed treatment effects should be interpreted with caution, as residual confounding and ecological bias cannot be fully excluded. Nevertheless, it is important to emphasize that the primary endpoints were derived exclusively from randomized controlled trials, which represent the highest level of evidence and substantially mitigate the risk of systematic bias. This strengthens the validity of the pooled estimates and supports the robustness of the overall findings.

Looking ahead, future research should prioritize large-scale randomized trials to enable a more granular assessment of treatment effects and to better identify patient subgroups most likely to benefit from TNK. In particular, further investigations are needed to clarify the interaction between TNK efficacy and the availability, timing, and success of EVT, as well as to determine the optimal selection criteria based on advanced imaging and clinical profiles. Additionally, efforts should be directed toward refining and standardizing treatment algorithms within the extended therapeutic window, with the goal of maximizing functional outcomes while maintaining safety across diverse healthcare settings.

Overall, this analysis supports a tailored approach to reperfusion therapy in acute ischemic stroke, highlighting TNK as a valid option, especially in non-EVT settings or resource-limited environments.

In conclusion, this meta-analysis demonstrated that TNK used between 4.5 h and 24 h is associated with improved rates of both good and excellent functional outcomes at 90 days compared with BMT, with a more pronounced benefit observed in patients treated with IVT alone, rather than those undergoing EVT plus IVT. In addition, TNK was associated with higher rates of recanalization, reflecting a greater proportion of saved ischemic penumbra as assessed by perfusion imaging. From a safety perspective, although a higher numerical incidence of sICH was observed in the TNK group, this did not reach statistical significance, and 90-day mortality rates were comparable between the two groups.

Overall, these findings support TNK as an effective option for acute reperfusion therapy in ischemic stroke, with potential advantages over the BMT within the extended therapeutic window of up to 24 h from symptom onset.

## Figures and Tables

**Table 1:** Baseline characteristics of 7 RCT studies included in the meta-analysis [18,19,20,21,22,23,24].

**Title**	DOI	Author	Year	Study Design	Subgroups	EVT Permitted	Baseline NIHSS, Median (IQR)	Ischemic Core Volume	Clinical Outcome
Efficacy of Tenecteplase in Large Vessel Occlusion Stroke Within 24 Hours of Symptom Onset: The ETERNAL-LVO Randomized Controlled Trial	https://www.ahajournals.org/doi/10.1161/STROKEAHA.125.052511	Yogendrakumar et al.	2025	RCT	Tenecteplase vs. standard of care	no	13 (7–19) vs. 14 (7–18)	5 mL (0–22) vs. 6 mL (0–18)	90 days mRS 0–1, symptomatic intracerebral hemorrhage
Tenecteplase for Acute Non–Large Vessel Occlusion 4.5 to 24 Hours After Ischemic Stroke The OPTION Randomized Clinical Trial	https://doi.org/10.1001/jama.2026.0210. PMID: 41642827; PMCID: PMC12878635.	Gaoting Ma et al.	2026	RCT	Tenecteplase vs. standard of care	no	7 (5–9) vs. 6 (5–9)	0 mL (0–3.7) vs. 1 mL (0–4.2)	90 days mRS 0–1
Intravenous Tenecteplase for Acute Ischemic Stroke Within 4.5–24 Hours of Onset (ROSE-TNK): A Phase 2, Randomized, Multicenter Study	https://doi.org/10.5853/jos.2023.00668. PMID: 37608533; PMCID: PMC10574303.	Wang et al.	2023	RCT	Tenecteplase vs. standard of care	no	7.50 (6.00–10.75) vs. 7.00 (6.00–8.75)	0.32 mL (0.00–2.28) vs. 0.40 mL (0.09–1.48)	90 days mRS 0–1, symptomatic intracerebral hemorrhage
Tenecteplase for Stroke at 4.5 to 24 Hours with Perfusion-Imaging Selection - TIMELESS	https://doi.org/10.1056/NEJMoa2310392. PMID: 38329148.	Albers et al.	2024	RCT	Tenecteplase vs. placebo	yes	12 (8–17) vs. 12 (8–18)	<20 mL 163 vs. 184 20–50 mL 51 vs. 29 ≥50 mL 12 vs. 16	90 days mRS, Symptomatic intracerebral hemorrhage
Tenecteplase for Ischemic Stroke at 4.5 to 24 Hours without Thrombectomy- TRACE III	https://doi.org/10.1056/NEJMoa2402980. PMID: 38884324.	Xiong et al.	2024	RCT	Tenecteplase vs. standard of care	no	11 (7–15) vs. 10 (7–14)	16.4 mL (5.7–28.4) vs. 14.9 mL (6.0–29.3)"	90 days mRS 0–1, Symptomatic intracerebral hemorrhage, death
Tenecteplase Thrombolysis for Stroke up to 24 Hours After Onset with Perfusion Imaging Selection: The CHABLIS-T II Randomized Clinical Trial	https://doi.org/10.1161/STROKEAHA.124.048375. PMID: 39744861.	Cheng et al.	2025	RCT	Tenecteplase vs. best medical treatment	yes	9 (5–14) vs. 9 (6–16)	6.0 mL (2.0–25.0) vs. 9.0 mL (3.0–22.0)	Primary outcome: major reperfusion without sICH within 24 to 48 hours. Secondary outcomes: recanalization, infarct growth, major neurological improvements, hemorrhagic transformation within 24 to 48 hours, systemic bleeding at discharge, and mRS at 90 days.
Tenecteplase Plus Butyphthalide for Stroke Within 4.5–6 Hours of Onset (EXIT-BT): a Phase 2 Study	https://doi.org/10.1161/STROKEAHA.125.053256.	Chen et al.	2025	RCT	Tenecteplase Plus Butyphthalide vs. Butyphthalide	no	-	-	primary endpoint was sICH. Secondary endpoints included excellent functional outcome (mRS 0–1) at 90 days.

Abbreviations: RCT (randomized controlled trial), NIHSS (National Institutes of Health Stroke Scale), TNK (Tenecteplase), mRS (modified Rankin scale), sICH (symptomatic intracranial hemorrhage), IQR (Interquartile Range).

**Table 2:** Comparison of baseline characteristics by treatment group, TNK vs. BMT.

Variable	TNK Group (*n*. 973)	BMT Group (*n*. 967)	*p* Value
Age (years)	66.4 ± 3.6	66.5 ± 3.7	0.55
Gender (female)	362	369	0.76
NIHSS score at admission	8.5 ± 2.7	8.2 ± 2.6	0.01
Core volume (mL)	5.9 ± 6.6	6 ± 6	0.73
Penumbral volume (mL)	83.2 ± 35.2	85.9 ± 35.5	0.09

Abbreviations: NIHSS (National Institutes of Health Stroke Scale), TNK (tenecteplase), BMT (best medical treatment).

## Data Availability

The original contributions presented in this study are included in the article/Supplementary Materials. Further inquiries can be directed to the corresponding author(s).

## Supplementary Materials

The following supporting information can be downloaded at https://www.mdpi.com/article/10.3390/neurolint18060116/s1: Figure S1: Risk of bias assessment of the included randomized controlled trials according to the ROB2 tool; Figure S2: Forest plot showing the distribution of outcomes stratified by type of treatment; Figure S3: Forest plot of 90-day mortality comparing TNK and BMT. Mortality rates were similar between groups (11.2% vs 9.9%; OR 1.13, 95% CI 0.84–1.52; *p* for heterogeneity = 0.78); Figure S4: Forest plot of sICH comparing TNK and BMT. A higher incidence was observed in the TNK group, although the difference was not statistically significant (3.1% vs 1.3%; OR 2.03, 95% CI 0.97–4.26; *p* for heterogeneity = 0.37); Table S1: PRISMA 2020 Checklist [28].
