# Supplementary figures and images for: Tenecteplase With or Without Mechanical Thrombectomy in Acute Ischemic Stroke at 4.5 to 24 h: An Updated Meta-Analysis of Randomized Controlled Trials
Source: Neurol Int. 2026 Jun 11;18(6):116. doi: 10.3390/neurolint18060116 (PMC13304984; doi:10.3390/neurolint18060116)

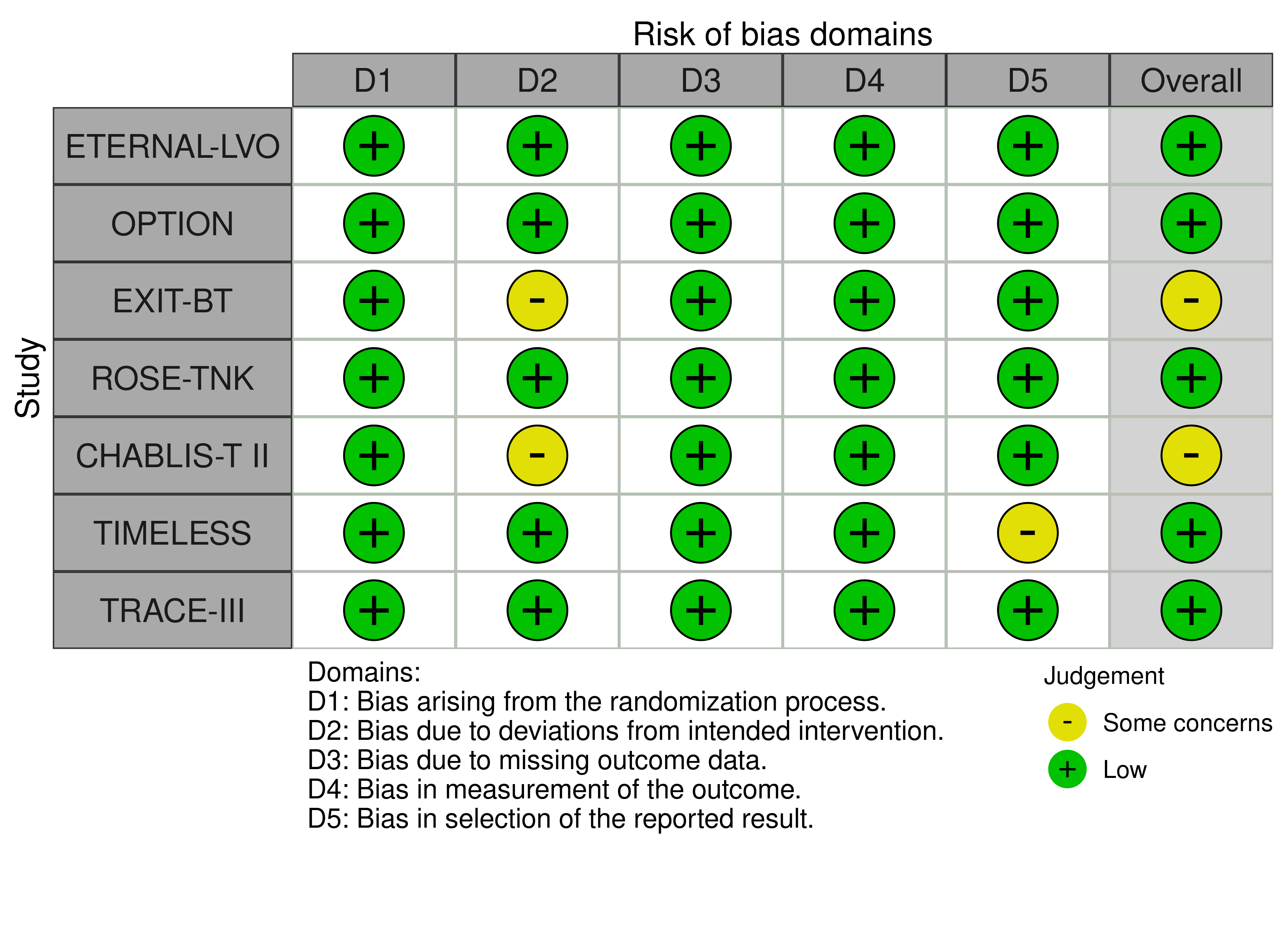

Supplement: Supplementary file 1 [file neurolint-18-00116-s001.zip › neurolint-4291786-supplementary - for proof/Figure S1.png]

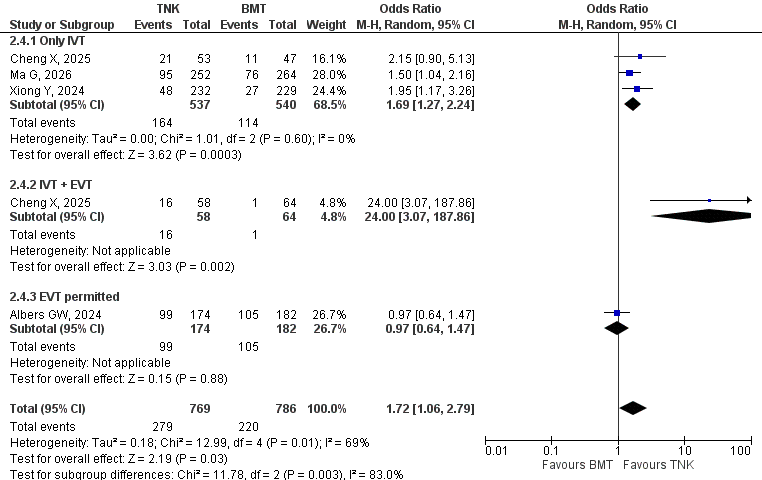

Supplement: Supplementary file 1 [file neurolint-18-00116-s001.zip › neurolint-4291786-supplementary - for proof/Figure S2.png]

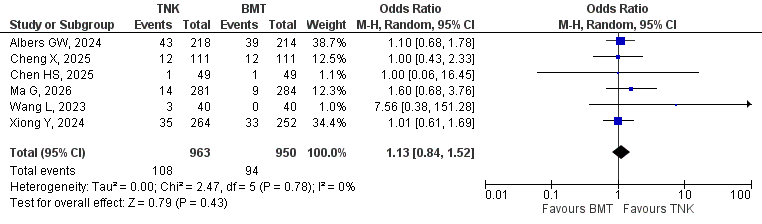

Supplement: Supplementary file 1 [file neurolint-18-00116-s001.zip › neurolint-4291786-supplementary - for proof/Figure S3.png]

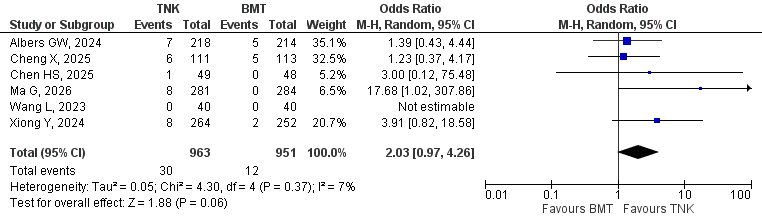

Supplement: Supplementary file 1 [file neurolint-18-00116-s001.zip › neurolint-4291786-supplementary - for proof/Figure S4.png]
